# Milk Oral Lyophilizates with Loratadine: Screening for New Excipients for Pediatric Use

**DOI:** 10.3390/pharmaceutics14071342

**Published:** 2022-06-24

**Authors:** Sonia Iurian, Cătălina Bogdan, Ștefana Suciu, Dana-Maria Muntean, Lucia Rus, Mihaela Berindeie, Szidonia Bodi, Rita Ambrus, Ioan Tomuță

**Affiliations:** 1 Department of Pharmaceutical Technology and Biopharmacy, Faculty of Pharmacy, “Iuliu Hațieganu” University of Medicine and Pharmacy, 41 V. Babes Street, 400012 Cluj-Napoca, Romania; sonia.iurian@umfcluj.ro (S.I.); suciu.stefana@umfcluj.ro (Ș.S.); dana.muntean@umfcluj.ro (D.-M.M.); mihaelaberindeie95@gmail.com (M.B.); bodi_szidonia@yahoo.com (S.B.); tomutaioan@umfcluj.ro (I.T.); 2 Department of Dermopharmacy and Cosmetics, Faculty of Pharmacy, “Iuliu Haţieganu” University of Medicine and Pharmacy, 12 I. Creangă Street, 400010 Cluj-Napoca, Romania; 3 Department of Drug Analysis, Faculty of Pharmacy, “Iuliu Haţieganu” University of Medicine and Pharmacy, 6 Louis Pasteur Street, 400349 Cluj-Napoca, Romania; lucia.rus@umfcluj.ro; 4 Faculty of Pharmacy, Institute of Pharmaceutical Technology and Regulatory Affairs, University of Szeged, Eotvos u. 6, H-6720 Szeged, Hungary; ambrus.rita@szte.hu

**Keywords:** milk, freeze-dried orodispersible tablets, design of experiments, pediatric dosage form

## Abstract

The development of suitable formulations for the pediatric population remains a challenging field with great advances reported every year in terms of excipients and technology. When developing pediatric formulations, the acceptability of medicines represents a key element to consider. For this reason, milk can be a widely accepted excipient with taste-masking properties and supplementary advantages for drug solubility. In recent years, the orodispersible dosage forms have come onto the market as child-friendly formulations. The current study aimed to develop freeze-dried orodispersible dosage forms containing bovine milk or infant formulae as the main component. In the first stage, an exploratory study evaluated the mechanical properties of placebo milk formulations and the suitability of milk as a matrix-forming agent. As the appropriate mechanical strength to withstand manipulation was demonstrated, milk oral lyophilizates were loaded with a poorly soluble model API, loratadine. Hence, a D-optimal design was conducted to prepare milk lyophilizates with loratadine and to evaluate the effects of three factors (dose of loratadine, the lyophilizate size, and the type of milk) and their interactions. Finally, three formulations were prepared to confront the predictions of the DoE and further studied to thoroughly understand the observed effects. The experimental results showed the potential of milk in the development of oral lyophilizates loaded with different doses of suspended API.

## 1. Introduction

In recent years, there has been an important effort toward developing optimal age-appropriate formulations. In the early 2000s, the high prevalence of off-label unauthorized drug product prescriptions in the pediatric population drew the attention of regulatory authorities. They noticed the scarcity of adequate medication tailored to the demands of each age group regarding the high diversity of this patient population, ranging from neonates to adolescents. Since then, global stakeholders, including academia, industry, and regulatory agencies, have worked together to fill the therapeutic gaps with the most adequate formulations based on the currently available technologies [1,2,3]. To facilitate the patient’s compliance, it is important to consider those attributes affecting the acceptability of the formulation, such as palatability, frequency of administration, or administration complexity [4]. The development of medicines for the pediatric population is particularly challenging. Emphasis is placed upon technologies and dosage forms intended to improve current formulations in terms of ease of administration, patient compliance, and safety profile [5,6]. In this context, orodispersible dosage forms have reached the pharmaceutical market due to their easy administration that overcomes swallowing difficulties and emerged as dosage forms that fit the needs of special groups of patients, such as the elderly and children [7]. They showed important benefits over the traditional dosage forms in terms of patient preference and acceptability [8]. Among those, the oral lyophilizates (OLs) obtained by freeze drying drug solutions or suspensions display light structures that disintegrate rapidly in the oral cavity, obtained with a small number of excipients but with high frailty that often impairs manipulation [9]. In the preparation of OLs, the two essential classes of excipients that determine the strength and stability of the structures are the fillers and the matrix-forming agents. Fillers, such as mannitol, glucose, trehalose, or lactose, were reported to give satisfactory results in terms of strength and disintegration [10], as well as polymeric matrix-forming agents, such as methylcellulose, xanthan gum, polyvinyl acetate [9,11,12], gelatin, and amino acids [13,14]. Furthermore, the suitable selection of the filler is correlated with the mouthfeel attributes [15]. When the pediatric population is targeted, a rigorous, benefit vs. risk approach is advised for the selection of excipients, and researchers have started looking for excipients with improved safety and acceptability profile [4]. Out of all the foods, milk is the most widely accepted by children, and its use as a liquid for drug administration has already been confirmed [16]. Rich in carbohydrates, proteins, and fats, stabilized in the form of a complex solution–emulsion–suspension system, it has been studied as an excipient, while its constituents (e.g., casein) as drug delivery systems. The increased tolerance and acceptability, the pleasant taste, the high availability, and the low associated costs recommend it as useful in the preparation of medicines. Milk from various sources has already been tested; bovine milk has been used as such in liquid formulations [17,18,19], freeze-dried [20], spray-dried milk [21,22], and infant formulae [5,20,23] for solid dosage forms, with important benefits for drug solubility. As manufacturing technologies, direct compression was used to transform powdered milk into tablets, dispersible tablets, or minitablets [5,20,23] and spray drying of API milk dispersions for the preparation of powders [21,22], with promising results, especially when associated with traditional excipients. A recent literature review focused on milk formulations highlighted how active substances can be incorporated into milk constituents and the further impact on solubility and bioavailability [24]. As far as the stability of such systems is concerned, freeze drying was reported to be the method that best preserves the properties of milk [25]. Lal and coworkers described the preparation of OLs loaded with two antiretrovirals containing 8.8% of dry milk in association with mannitol, carboxymethyl cellulose, and Tween 80 [2]. However, information on the contribution of milk to the formation of the porous structure, to the disintegration of the solid form, and to the dissolution of the active substances in OLs is limited. Up to this point, to the best of the authors’ knowledge, no work has been published on the potential of milk as the main excipient in the preparation of OLs.

The main questions that this study aims to answer are whether lyophilized milk could be a sufficiently resistant matrix to handle and whether it could incorporate suspended insoluble active ingredients. Among the secondary objectives of the study were evaluating the influence of the type of milk on the critical quality characteristics of the dosage forms, the impact of the dose of active pharmaceutical (API) ingredient on the lyophilized structure, and that of the alveolae volume or the lyophilizate size on the previously mentioned characteristics. The choice of API was based on the increased frequency of allergic manifestations in children frequently treated with loratadine, as a representative of the anti-H1 antihistamines listed in the WHO List of Essential Medicines for Children [26]. Loratadine is efficient in low doses, requires age-related dose adjustment, and has low aqueous solubility, which makes it a good candidate to test the performance of milk freeze-dried cakes.

The study is divided into three parts, the preliminary part of which was meant to evaluate the mechanical properties of placebo milk formulations. Subsequently, based on the favorable results of the mechanical tests, lyophilizates loaded with loratadine as a model substance were prepared, according to a design of experiments in which the effects of three factors (dose of loratadine, the volume of the alveolae, or lyophilizate size and the type of milk) were studied. Finally, three formulations were prepared to confront the predictions of the design of experiments and were characterized for an in-depth understanding of the observed effects.

## 2. Materials and Methods

### 2.1. Materials

The active pharmaceutical ingredient (API) loratadine was purchased from Quimica Sintetica (Madrid, Spain). The types of milk coded L1 (skimmed milk, 1.5% fat) and L2 (full-fat milk, 3.5%) were locally produced by Albalact (Bucharest, Romania), while L3 (NAN A.R.) and L4 (NAN 1 Optipro) were infant formulae purchased from Nestle (Bucharest, Romania).

### 2.2. Preliminary Study to Investigate the Mechanical Properties of the Freeze-Dried Milk

Four types of milk, coded L1, L2, L3, and L4, with the composition described in Table 1, were included in the preliminary study that aimed to test whether milk could yield freeze-dried structures with mechanical profiles that allow easy and safe handling. L1 and L2 were used as such, while L3 and L4 were reconstituted following the recommendations of the producer. All four samples were poured into 0.2, 0.5, and 1 mL blister alveolae using an automatic pipette, submitted to freeze drying (see Section 2.3), and then evaluated for the mechanical profile (see Section 2.6.2).

### 2.3. Freeze Drying

The samples were subjected to a freeze-drying process (VirTis Advantage Plus, SP Scientific, Gardiner, NY, USA) consisting of a 12 h freezing stage at −55 °C, followed by 24 h primary drying at −25 °C and 150 mTorr, and 10 h secondary drying at 20 °C and 300 mTorr.

### 2.4. Design of Experiments

The DoE methodology is a statistical tool that allows the rational planning of experiments to cover as wide a study domain as possible [27]. The study was performed using a D-optimal screening experimental design, with three independent variables and three variation levels, developed using Modde 13 software (Sartorius Stedim, Umeå, Sweden). According to the DoE, 16 runs and 3 replicated center points were generated, as shown in Table 2.

The loratadine content was chosen as a quantitative factor (X1) and varied on three levels: 0–5–10 mg per OL. The suspension volume poured into each blister pocket was the second quantitative factor (X2) and varied between 0.2, 0.5, and 1 mL. Four types of milk were used as a qualitative factor (X3), which were encoded as L1 (1.5% fat), L2 (3.5% fat), L3, and L4, with compositions described in Table 1.

The selected responses entailed attributes related to a pharmaceutical product’s quality, efficacy, and safety. They consisted of: disintegration time (Y1), mechanical properties (Y2—hardness; Y3—rigidity at 1 mm depth; Y4—fracturability), dissolution profile (Y5—% of dissolved loratadine after 5 mi; Y6—% of dissolved loratadine after 10 min), and particle size analysis (Y7—average particle size; Y8—PDI).

The same software (Modde 13, Sartorius Stedim, Sweden) was employed to model DoE data. In this sense, data processing, fitting, and statistical parameters calculation were performed, and the effects of the variables on the selected responses were graphically represented as coefficient histograms and response surfaces. The ANOVA test (variance analysis) was applied for all the responses to evaluate the validity of the experimental design. The model interpretation was carried out based on the regression equation coefficients. Their signs indicate the type of influence (negative or positive) they exerted on the response, while the absolute value shows the magnitude of the effect.

### 2.5. OLs Preparation

Placebo OLs were obtained by pouring 0.2, 0.5, or 1 mL of milk into the blister pockets according to the data presented in Table 2. For the OLs containing API, loratadine was suspended into the selected type of milk up to an amount of 5 mg/volume unit or 10 mg/volume unit. For each formulation, 30 mL suspension was obtained, and the exact volumes of 0.2, 0.5, or 1 mL were poured into 30 blister alveolae (3 blisters × 10 alveolae for each formulation). Immediately after the transfer into the blister alveolae, the samples were frozen at −80 °C to prevent API sedimentation.

### 2.6. OLs Pharmaceutical Characterization

#### 2.6.1. Disintegration Time

The disintegration time was measured according to the method described in the *European Pharmacopoeia* [28]. Six samples from each formulation were placed in 200 mL distilled water and kept at 20 ± 5 °C; the necessary time to fully disintegrate was recorded using a digital stopwatch. The mean disintegration time (Y1) and standard deviation were calculated for each formulation.

#### 2.6.2. Mechanical Characterization

Texture analysis is a common method of evaluating lyophilized matrices due to its ability to record small variations in the force or load required to penetrate the freeze-dried cake up to a given distance [29]. The characterization of the OLs’ mechanical properties was performed by using a CT3 texture analyzer (Brookfield Engineering, Middleborough, MA, USA). For this purpose, a simple compression test was carried out by applying a load of 10 g and a speed of 0.10 mm/s to press the freeze-dried material until deformation of 2 mm for 0.2 mL OLs or 3 mm for 0.5 mL and 1 mL OLs was reached. The OLs’ texture parameters describing the resistance of the sample to the applied pressure and the load vs. distance graphical curves were acquired with TextureProCT V 1.9 software. For each formulation, three OLs were tested, and the average and standard deviation were considered for the following parameters: hardness (Y2), rigidity (Y3), and fracturability (Y4).

#### 2.6.3. In Vitro Dissolution

The in vitro dissolution study was performed in compliance with the method described in the *European Pharmacopoeia* [28], using the dissolution tester equipped with rotating paddles (Pharma Test PT-DT 7, PTWS100, Hainburg, Germany). The dissolution study was carried out in 900 mL phosphate buffer, pH 6.8, at a stirring rate of 50 rpm at 37 °C. Sampling was performed after 5, 10, 15, 20, 30, and 35 min, each redrawn sample being replaced with the same volume of fresh dissolution media. The dissolved API was quantified with an HPLC method; chromatographic separation was achieved on a Phenomenex Luna C18 column (150 × 4.6 × 5 mm), maintained at 30 °C; the mobile phase was H_3_PO_4_ 0,1N: acetonitrile 60:40 (*v/v*). The flow rate was 1.5 m/min, and the injection volume was 50 µL. UV detection was performed at 250 nm [30]. The detected ratios of dissolved loratadine after 5 min (Y5) and 10 min (Y6) were included in the DoE. The mean values of the loratadine ratio and standard deviation were calculated out of three measurements.

#### 2.6.4. Particle Size Analysis of the Reconstituted Samples

The average hydrodynamic diameter (Z-average) (Y7) and polydispersity index (PDI)(Y8) were measured with the dynamic light scattering (DLS) technique using a Malvern Zetasizer Nano ZS90 (Malvern Instruments, Worcestershire, UK). The reported results are the mean of three determinations and the standard deviation.

### 2.7. Contact Angle Measurements (Wetting Properties)

The determination of the polarity was performed by using Dataphysics OCA 20 analyzer (Dataphysics Inc. GmbH, Filderstadt, Germany). Briefly, 0.10 g of each sample was compressed at 1-ton compression force with the hydraulic press (Perkin Elmer, Waltham, MA, USA). Three pastilles per sample were analyzed by dripping the surface of the sample with distilled water and diiodomethane. The change of the contact angle (Θ) in a time interval of 1–25 s was measured, and the values of the contact angle were determined according to the following calculation: surface free energy $\left(\gamma_{s}\right)$ consisting of a polar $\left(\gamma_{s}^{d}\right)$ and a dispersed part ($\gamma_{s}^{p})$, therefore, $\left(\gamma_{s}=\gamma_{s}^{d}+\gamma_{s}^{p}\right)$. It was obtained from the Wu-equation:

$$\left(1+\cos\Theta \right)\gamma_{l}=\frac{4\left(\gamma_{s}^{d}\gamma_{l}^{d}\right)}{\gamma_{s}^{d}+\gamma_{l}^{d}}+\frac{4\left(\gamma_{s}^{p}\gamma_{l}^{p}\right)}{\gamma_{s}^{p}+\gamma_{l}^{p}}$$

where Θ = contact angle; γ = surface free energy; s = solid phase; l = liquid phase; d = dispersion component; p = polar component, based on the values of the surface tension of the applied liquids, $\left(\gamma_{l}=\gamma_{l}^{d}+\gamma_{l}^{p}\right)$, reported in the literature: $\gamma^{p}\;$= 50.2 mN/m, $\gamma^{d}\;$= 22.6 mN/m for distilled water and $\gamma^{p}\;$= 1.8 mN/m, $\gamma^{d}\;$= 49 mN/m for diiodomethane [31].

### 2.8. Solid-State Analysis

#### 2.8.1. X-ray Powder Diffraction Analysis (XRPD)

XRPD was used to investigate the physical state of the API in the different stages of the preparation process (for LOR and the OLs). XRPD spectra were collected with a BRUKER D8 Advance X-ray diffractometer (Bruker AXS GmbH, Karlsruhe, Germany) system with Cu Ká1 radiation (ë = 1.5406 Ĺ) over the interval 5–30°/2. The experimental setup was the following: target, Cu; filter, Ni; voltage, 40 kV; current, 40 mA; time constant, 0.1 s; angular step, 0.010. The total area of the characteristic three peaks with the largest intensity was examined for the determination of the degree of crystallinity, after smoothing and background removal.

#### 2.8.2. Fourier-Transform Infrared Spectroscopy (FT-IR)

FT-IR spectra of the OLs were obtained by using Fourier-transform infrared spectroscopy (Thermo Nicolet AVATAR 330, Thermo Electron, Waltham, MA, USA) equipped with a deuterated triglycine sulfate detector. The samples were ground with 150 mg potassium bromide, and the mean values of 128 co-added scans at a resolution of 4 cm^−1^ over 4000–400 cm^−1^ wavenumber region were collected and interpreted using the GRAMS/AI Version 7.00 software.

#### 2.8.3. Scanning Electron Microscopy (SEM)

Scanning electron microscopy micrographs of the samples were acquired with Hitachi S4700 equipment (Hitachi Scientific Ltd., Tokyo, Japan) at an electric potential of 10 kV. The samples were coated with a sputter coater (Bio-Rad SC 502, VG Microtech, Uckfield, UK), and the OL’s microstructure was investigated.

## 3. Results and Discussion

Milk has long been explored as an excipient for pediatric dosage forms, either in a dry state for solids preparation or as such for liquid dispersions. However, to the best of the authors’ knowledge, its use as a multiple-function excipient for OL preparation is yet to be understood.

As one of the key issues in ensuring proper OL quality is overcoming the high friability, this study first focused on the selection of appropriate types of milk, which would grant the mechanical properties required for product transport, manipulation, and ejection from blister sockets without risking material loss, thus API dose inaccuracy.

### 3.1. Investigation of the Mechanical Properties of the Freeze-Dried Milk

**Figure 1:**
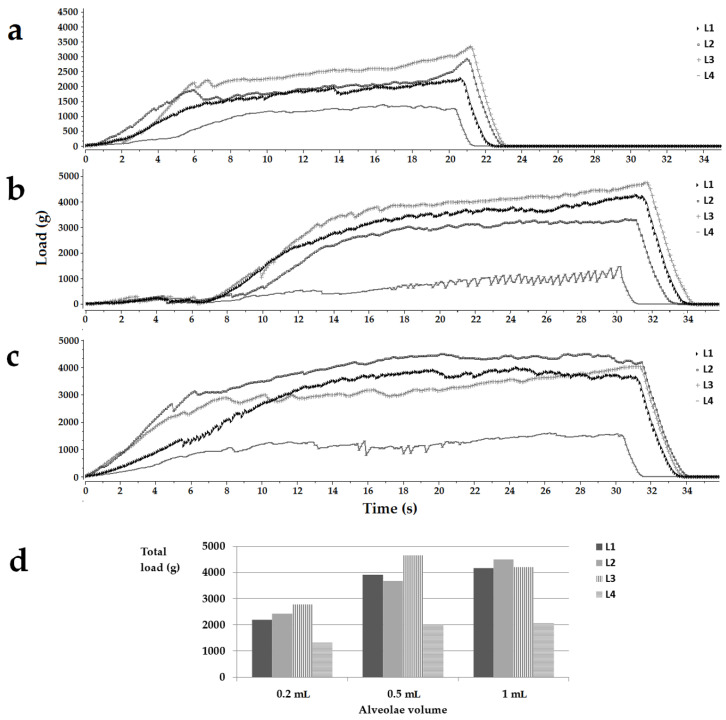
Load (g) vs. time(s) texture analysis representative profiles for the placebo OLs obtained in 0.2 mL (**a**), 0.5 mL (**b**), and 1 mL (**c**) alveolae and a histogram summarizing the total load of the samples (**d**). Legend: L1, L2, L3, L4, the type of milk.

The study’s first phase was testing the feasibility of using milk as a lyophilized matrix. Oral lyophilizates are known for their high porosity, leading to increased friability and low handling resistance. Because the strength of a lyophilized matrix depends on the formulation but also its size, lyophilized products were obtained from the four types of milk (L1, L2, L3, and L4) poured into blister sockets with different volumes, then extracted from alveolae and subjected to a compression test. The mechanical characteristics of the freeze-dried milk samples expressed as load vs. time curves are shown in Figure 1. The profiles below show the load encountered by the probe when descending at a constant speed into the sample due to the compaction of the solid material. The increase in the load with time, respectively, with the distance covered in the freeze-dried material is evident for all tested samples (Figure 1a–c). The depth of descent of the probe (displacement) was different depending on the product thickness correlated to the volume of the alveolae. For the products obtained in 0.2 mL alveolae, the displacement was 2 mm, while for thicker products obtained in 0.5 mL, respectively, 1 mL alveolae, the displacement was set to 3 mm. This explains the different profile lengths shown in Figure 1a compared to those in Figure 1b,c. The profiles also show sudden drops of load due to the fractures of the pore walls, which can be regarded as indicators of the brittleness of the structure [29]. The total hardness of the sample is estimated to be the maximum load reached during a compression cycle, and the highest hardness was reached for lyophilizates with volumes of 0.5 mL and 1 mL.

In the case of samples poured into 0.2 mL alveolae, the hardness decreased in the following order L3 > L2 > L1 > L4. For the 0.5 mL cakes, the highest hardness was obtained for the L3 samples, followed by L1, L2, and L4. In the case of samples with a volume of 1 mL, L2 milk led to the most resistant structures, followed by L1, L3, and L4 (Figure 1d). As far as the authors know, no results have been reported on the texture analysis of lyophilized milk, so studies involving pharmaceutical excipients were used for comparison. Casian et al. reported a minimum value of 300 g load for the oral lyophilizates to withstand easy handling, while tests on commercial oral lyophilizates revealed loads of less than 200 g up to 1100–1200 g [30,32]. Considering the total load achieved by the tested samples, it falls above the acceptable values mentioned in the literature, regardless of the sizes or the types of milk. Although the values reported in the literature correspond to products containing the traditional OL composition, the drugs associated with matrix-forming agents and fillers meant to reinforce the structures, even the weakest samples in this study, obtained with L4 milk recorded load values of more than 1317.00 ± 209.40 g, well over the acceptable limits. These results support the attempt to load the freeze-dried milk structures with an active pharmaceutical ingredient.

Up to this point of evaluating the lyophilized milk structures, the only variation pattern observed was the increase in load depending on the alveolae volume or solid size, with no evident dependence on the type of milk, which leads to the premise of possible interactions between the studied factors. Therefore, the study was continued with the implementation of a design of experiments able to highlight the interactions between factors and to quantify the contribution of factors to the total effect.

### 3.2. Design of Experiments (DoE)

DoE is based on the choice of those independent variables that can influence the quality of a product, in this case, the quality of oral lyophilizates, and their levels of variation, which will be the starting point for drawing up the DoE matrix. The products prepared according to the DoE matrix are characterized as completely as possible by determining a set of dependent variables. The goal of the DoE is to determine the regression models, which correlate the dependent variables coded with Y_1_ to Y_n_ with the independent variables coded with X_1_ to X_n_. Thus, equations such as Y_n_ = f(X_1_,...,X_n_)—reveals the influences of the independent variables and their interactions on the dependent variables. The models can be used for an in-depth understanding of the product and to further predict the characteristics of new products in the experimental domain.

As the characteristics of oral lyophilizates are multifactorial, and a sufficient resistance of the placebo structures does not guarantee the same behavior after loading an API, a DoE was developed to evaluate simultaneously the influences of the type of milk, the volume of the alveolae, and the API content, and their interactions. The dose of loratadine (X_1_) was the first quantitative variable for which two levels were assigned, 0 mg and 10 mg, and further, the software chose an intermediate value of 5 mg for the three replicate center points. Finally, the design matrix included experiments with 0 mg, 5 mg, or 10 mg loratadine per unit. These values were chosen according to the dose tapering recommendations for children between 2 and 12 years of age: 5 mg for those below 30 kg of body weight and 10 mg for those over 30 kg [33]. The alveolae volume (X_2_) was selected as the second quantitative factor, depending on the sizes that were easy to handle and that were considered acceptable for the administration to children. The aspect, sizes, and weights of OLs with different volumes were included in Table 3. The type of milk was set as qualitative variable X_3_ and varied on four levels, L1, L2, L3, and L4. The dependence of disintegration time on the lipid content was revealed in the literature for conventional tablets, so L1 and L2 bovine milk were chosen for the differences in fat. Two infant formulae (L3 and L4) were added to the design due to their standardized compositions that could grant easier approval as excipients, while L3 was selected for its higher viscosity that could ensure suspension homogeneity before freezing.

#### 3.2.1. Summary of Fit

Before interpreting the influence of independent variables on the responses based on the obtained mathematical models, the quality of the data fit was evaluated from statistical parameters, such as R2, Q2, validity, reproducibility, *p*-ANOVA, and lack of fit. The final mathematical models, whose performances are included in Table 4, were obtained following a fine-tunning stage of non-significant term elimination. For all responses, the variation in the data set was explained by the mathematical models (R2 > 0.671), and the prediction capacity of the models was good, with Q2 above 0.431 and close to R2. The chosen models were all valid (>0.25), and the data were recorded with high reproducibility, close to 1 for most of the responses.

#### 3.2.2. The Influence of Independent Variables on the Disintegration Time (Y1)

The disintegration time varied in a wide range, between 1.37 s and 265 s, and for some formulations even exceeded the 3 min acceptability limit of the *European Pharmacopoeia* for orodispersible tablets (Table S1). The disintegration time values were grouped at the two limits of the variation range; most of the values showed an ultra-fast disintegration in a matter of seconds, while others came close to 5 min. The floating tendency was also recorded for some of the formulations prior to wetting and disintegration. The mathematical model indicated that the most important effect came from the type of milk: bovine milk L1 and L2 led to slow disintegration, while L3 and L4, the infant formulae, determined fast disintegration, in less than 10 s. Within the four types of milk, the slowest disintegration was achieved by L2, the milk with 3.5% fat content, followed by L1, the milk with 1.5% fat content. As expected, the increase in fat content led to floating phenomena, longer wetting times, and slower disintegration. In contrast, infant formulae L3 and L4 led to ultra-fast disintegration, delayed in the L3 milk formulations by the viscosity agent found in the composition. Although the high-fat content of L2 milk was what caused the hydrophobicity of the matrices and a slower disintegration of L2 compared to L1, it was not so important with respect to the disintegration of OLs containing infant formulae. Although L2, L3, and L4 types of milk had almost similar fat content, L3 and L4 OLs disintegrated much faster than L2 OLs. This effect could be related to the lower ratios of saturated fats (Table 1) found in infant formulae that aim to mimic the fat composition found in human milk [34]. Another argument to explain the differences is the complex composition of infant formulae that sometimes reaches hundreds of substances, which are intended for easy reconstitution. L3 contains potato starch used to increase the viscosity of the formula to prevent regurgitation phenomena in newborns but also maltodextrin, which was reported to reduce the crystallinity of lactose and improve dispersibility and solubility of infant formulae [35]. On the other hand, L4 contains soy lecithin, an amphiphilic molecule that has been shown to improve the wetting properties of milk powders [36]. Similar differences were reported between the effects of bovine milk and infant formulae by Binte Abu Bakar et al. (2019) in studies on compressed dispersible tablets [20].

**Figure 2:**
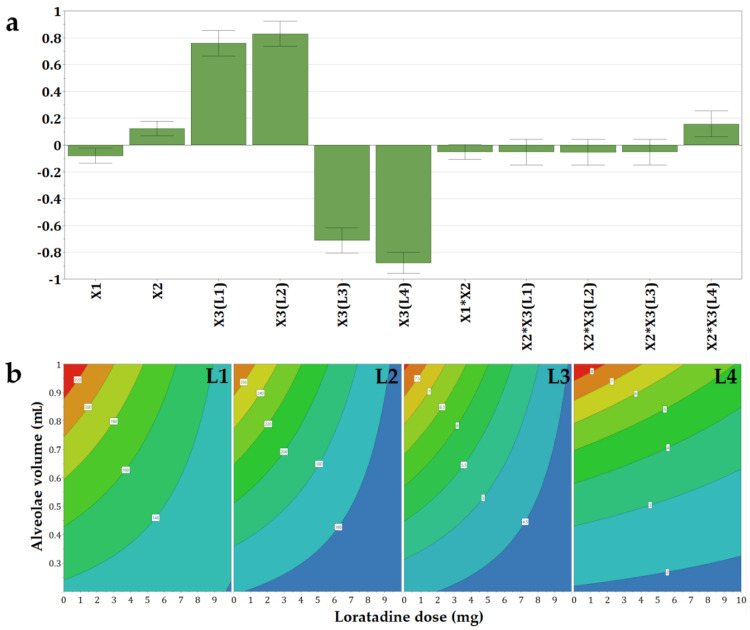
Coefficients of the regression equations displayed as (**a**) histograms and (**b**) representative contour plots for the disintegration time (Y1) as a function of the three independent variables.

The volume of the alveolae, and thus the size of the OL, had an important impact on disintegration, however, with a lower magnitude when compared to the type of milk (Figure 2a). Large OLs prepared out of high volumes of mixtures had high disintegration times. This effect was valid for placebo OLs. However, despite the increase in alveolar volume/OL size, the disintegration times were at their lowest for the OLs with 10 mg of loratadine.

Out of the four types of milk, the effect of loratadine content was barely noticed for L4, while the effect of the alveolae size was the most evident (Figure 2b). In contrast, the OLs containing L1, L2, and L3 milk showed a negative influence of the API dose. Thus, the increase in loratadine dose led to a faster disaggregation because insoluble loratadine was interposed between the constituents of the milk during drying, and in addition, an increase in loratadine content in the same volume of suspension was equivalent to a decrease in milk. The same effect of high milk content on disintegration was reported by Orubu and coworkers for compressed tablets [5] and by Pinto and coworkers for minitablets prepared with powdered milk and paracetamol [23].

#### 3.2.3. The Influence of Independent Variables on the Mechanical Properties (Y2–Y4)

**Figure 3:**
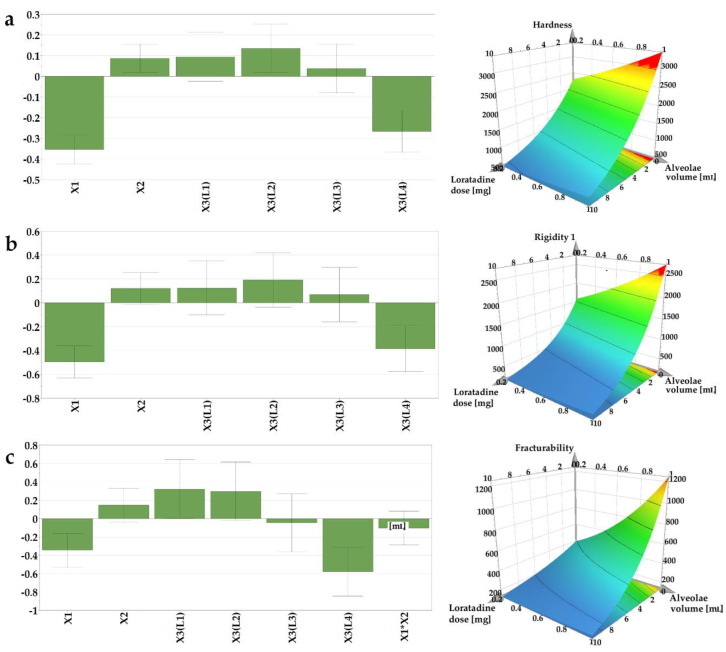
Coefficients of the regression equations displayed as histograms and representative response surfaces plotted for L1 milk for (**a**) hardness (Y2), (**b**) rigidity (Y3), and (**c**) fracturability (Y4).

The mechanical attributes of the OLs containing loratadine prepared according to the DoE matrix were determined to evaluate whether the structures containing API provide adequate mechanical resistance to prevent breaking of the structures during transport or handling. The texture profiles as load vs. time were recorded, out of which three parameters were determined: Y2—hardness as the total load at the final displacement; Y3—rigidity at 1 mm depth, and Y4—fracturability as the load that produces the first fracture. Out of the tested factors, the API and the L4 type of milk had a negative influence on the calculated mechanical parameters (Figure 3).

The hardness of the sample is a result of the intermolecular bonding force and the contact points between the ingredients [37]. Within texture assessment, the hardness (Y2) of the samples was measured as the force necessary to attain the target deformation selected for the particular size of OL. The recorded values varied between 289, 8 g (N14), and 4486 g (N7). The lowest hardness was obtained in the case of the smallest OLs (0.2 mL sample volume) containing L4 and the highest ratio of API (10 mg), in contrast with the sample N7, prepared with L2 (1 mL sample volume and without API), where the highest hardness value was recorded. In comparison with the placebo OLs, the addition of the API had a significantly negative impact on the sample mechanical resistance, leading to weaker structures. However, these results are above the lowest values reported by Vanbillemont 2020 for some marketed OLs, where the minimum values reported were less than 200 g [32]. During the freeze-drying process, the ice is replaced by pores, resulting in a highly porous matrix [32]. The total porosity of the OL impacts the hardness of the samples; a decrease in the porosity leads to an increase in contact points within the matrix-forming agents, which stiffens the structure, resulting in an increase in hardness [37].

On the contrary, in our case, the addition of API led to the brittleness of the samples due to the interspersion of the API within the network, creating more friable structures. These results are in agreement with the disintegration data that revealed faster disintegration of samples with high doses of loratadine.

The rigidity values (Y3), measured as the hardness of the sample at a penetration depth of 1 mm, ranged between 82.2 and 3735 g. These measurements, recorded at the same depth, regardless of the size of the OL, indicate the same variation as the samples with the smallest size (0.2 mL volume) prepared with L4 type of milk, and the highest ratio of API (10 mg) displayed the lowest values for rigidity, while the samples with the highest volume (1 mL), prepared with L2 type of milk and without API, had the highest rigidity.

The internal ruptures and fractures of the samples were recorded as the probe progressed to the pre-established target deformation of 2 or 3 mm. The load value at the first fracture was recorded as the fracturability (Y4) of the samples. This parameter gives information on the sample`s resistance to fractures, a lower value indicating a more brittle product. Consistent with the previous results, the lowest value (42.2 g) for fracturability was obtained for N14, while the highest value, 3320 g, was obtained for N7, considered the most resistant to fractures.

When comparing the upper results with others reported in the literature for OLs prepared with traditional excipients, milk as a freeze-dried matrix yielded comparable results with those obtained by Casian et al. (2017) for OLs with xanthan gum and mannitol, or by AlHusban et al. (2010) for OLs with gelatin and various saccharides [13,30].

#### 3.2.4. The Influence of Independent Variables on the Dissolved Loratadine after 5 and 10 min (Y5, Y6)

**Figure 4:**
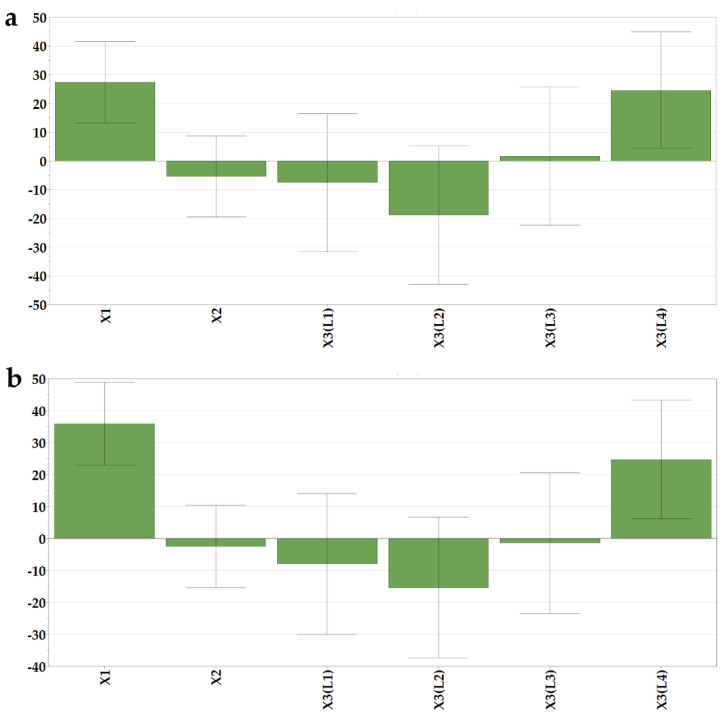
Coefficients of the regression equations displayed as histograms. (**a**) % of dissolved loratadine after 5 min (Y5) and (**b**) 10 min (Y6).

The dissolution behavior was assessed for 35 min, but out of the six sampling points, only two were included as responses in the experimental design, the % of dissolved loratadine after 5 and 10 min (Y5 and Y6), as after 10 min, the differences between the formulations flattened out, and no significant variations could be identified. The effect of the loratadine content was shown (Figure 4) with a significantly positive influence on the released percentages in the first part of the study. Its magnitude increased at the second time point, as dissolution unfolded. The increase in loratadine dose was shown to reduce the strength of the structures and the disintegration times. Moreover, higher doses of loratadine coincide with lower milk content per tablet, and in the case of bovine milk, this has an important impact on hydrophobicity and thus on API dissolution. Orubu also showed the negative effect of high ratios of milk on the dissolution rates of API [5].

The type of milk also had a significant effect on the % of dissolved loratadine. L4 milk led to faster dissolution. L4 milk OLs were also those with the shortest disintegration times, which granted fast contact of the API with the media and subsequent dissolution. Additionally, the presence of soy lecithin as an emulsifier in the L4 infant formula could have determined the fast dissolution of loratadine from those formulations. Although L3 milk OLs also displayed fast disintegration times, in terms of seconds, the effect of L3 milk on dissolution was not significant.

#### 3.2.5. The Influence of Independent Variables on Particle Size and Polydispersity Index (Y7, Y8)

The particle size determination was meant to give some hints regarding the aggregation phenomena that might have occurred during processing and the further palatability of the product. The average particle sizes of the reconstituted dispersions ranged between 292.97 ± 6.67 nm (N3) and 665.83 ± 34.90 nm (N9).

**Figure 5:**
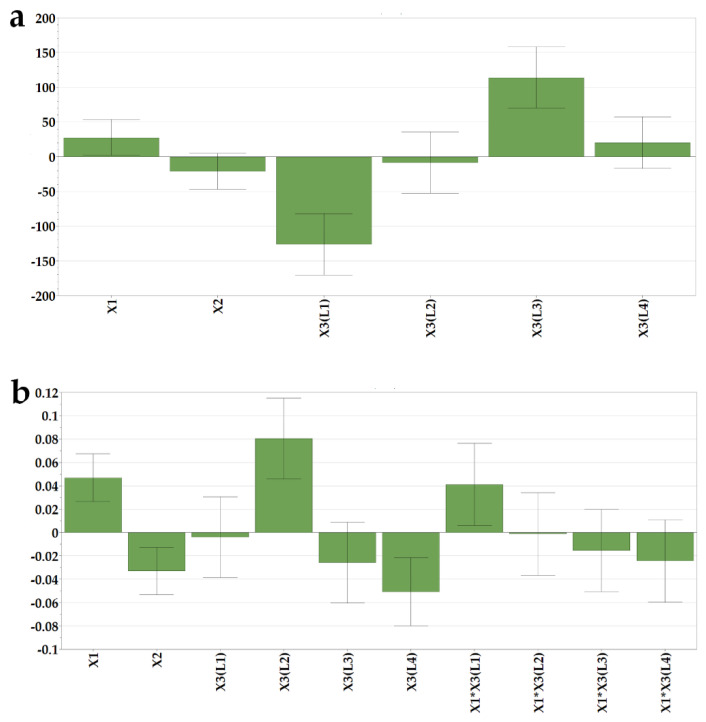
Coefficients of the regression equations are displayed as histograms and representative response surfaces for (**a**) particle size (Y7) and (**b**) polydispersity index (Y8).

As shown in the histogram of the regression coefficients (Figure 5a), the highest particle sizes were obtained for formulations with high drug loading and associated with the L3 milk. On the contrary, the use of bovine milk L1 led to decreased particle sizes of the reconstituted samples.

As loratadine was incorporated into the initial suspensions as a micronized powder, with D50 of 3 µm and D90 of 7 µm, and milk constituents were also present in the reconstituted dispersions, the polydispersity index increased with the higher drug load. Additionally, an increase in polydispersity was obtained when L2 milk was used as a freeze-dried matrix-forming agent due to its higher lipid content.

No aggregation phenomena were recorded in milk constituents during freeze drying, as there were no significant differences between the average particle sizes of reconstituted placebo OLs and milk samples in their initial liquid state. This could be an argument in favor of freeze drying as a technique to obtain solid milk dosage forms, as the aggregation phenomena with an increase in particle size after redispersion were reported for compressed tablets [20]. The acceptability of dispersible solids is influenced by many criteria among which is the particle size after dispersion in the saliva. The acceptability limit for granule sizes within orodispersible tablets was 244 µm; therefore, all the OL formulations prepared in this study are well within this range [38].

**Figure 6:**
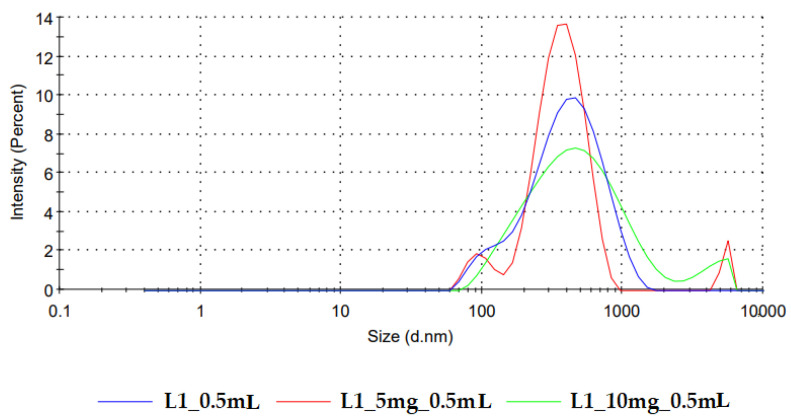
Particle size distribution of freeze-dried reconstituted samples of L1 milk and L1 milk loaded with increasing doses of loratadine.

The particle size distribution obtained for the reconstituted placebo formulations coincides with the DLS footprint reported by Kytariolos et al. Figure 6 displays the representative curves of particle size distribution for reconstituted freeze-dried L1 milk prepared in medium-sized alveolae compared to the same product loaded with increasing doses of loratadine of 5 mg and 10 mg, respectively. Bimodal distributions were obtained, with the first peak between 60 and 150 nm attributed to casein micelles and a second one between 200 and 800 nm corresponding to the fat globules [18].

The addition of loratadine led to the appearance of a third peak in the micrometer range that corresponds to the API. This correlates with the average size increase with the API dose revealed by the model coefficients and is in line with the variation of the polydispersity index. DoE analysis confirmed these observations; the PDI increased with the loratadine dose (Figure 5b) and L2 milk and decreased with the higher alveolae volumes, resulting in higher milk ratios and L4 milk. The narrow distribution obtained for L4 milk could be related to the excipients and other insoluble compounds found in the infant formula that display the same sizes as milk lipids, which led to low PDI correlated with particle sizes at the upper limits of the measured variation range.

### 3.3. DoE Conclusions

The DoE revealed the most important effects the three studied factors had on the OLs’ pharmaceutical characteristics:

- bovine milk (L1 and L2) and large volumes of alveolae prolong disintegration, while infant formulae and high doses of API ensure rapid disintegration;
- the use of L2 milk and high-volume alveolae increase the resistance of oral lyophilizates, while high doses of loratadine and the use of L4 milk raise the structure friability;
- two types of milk simultaneously meet the criteria of fast disintegration and appropriate mechanical profile: bovine milk L1 and infant formula L3;
- high API doses and the use of L4 milk led to the highest amounts of dissolved loratadine.

Although all responses in the DoE showed acceptable predictability, three formulations were chosen from the experimental domain to test the predictions of the DoE. Based on the observed effects, in the third part of the study, we chose to use two types of milk, L1 and L3, with increasing doses of loratadine, prepared in medium-sized alveolae. Table 5 shows the three formulations, their codes, predicted values, experimental values, and the calculated residuals.

The predictions were met for all parameters, except for the texture-analysis-derived parameters for the formulation L1_5 mg_0.5 mL due to the rather high variability between replicate samples or to the presence of nonlinear effects that were not accurately estimated by this type of DoE.

### 3.4. Supplementary Characterization of the OLs

The next step of the research was to submit those three formulations selected from the experimental domain to additional analyses for a thorough characterization and in-depth understanding of the observed effects.

#### 3.4.1. Wetting and Dissolution

**Figure 7:**
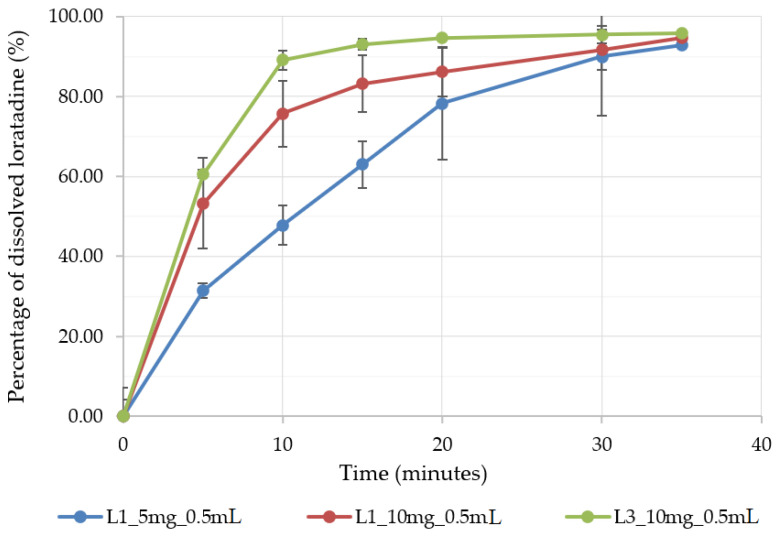
The dissolution profile of three selected OL formulations prepared in 0.5 mL alveolae.

According to the biopharmaceutical classification system, loratadine is a class II drug with low solubility and high permeability. The bioavailability depends on its luminal dissolution, the limiting step of intestinal absorption. Loratadine is also known as an API with pH-dependent solubility. Its weak base character determines the higher solubility of the ionized form in acidic media when compared to its solubility in neutral media [39]. As the fast dissolution of raw loratadine and commercial immediate-release formulations in acidic media were proved by several authors [40], an improved formulation would grant fast and reproducible dissolution at a higher pH. Therefore, the OL formulations were tested for in vitro dissolution at a pH of 6.8. Figure 7 depicts the dissolution profiles of three of the OL formulations prepared in medium-sized alveolae of 0.5 mL, two of them with L1 milk and increasing doses of API, of 5 mg (L1_5 mg_0.5 mL) and 10 mg (L1_10 mg_0.5 mL), and a formulation with L3 milk and a dose of 10 mg of loratadine (L13_10 mg_0.5 mL). As predicted by the DoE models, the increase in the API dose led to significantly higher dissolved API percentages, probably explained by the faster disintegration induced by the lower mechanical strength and by the lower milk content associated with the API dose increase. Out of the three formulations, the best dissolution profile was obtained for L13_10 mg_0.5 mL, with 80% of dissolved API in less than 10 min due to its good wettability and ultra-fast disintegration. The dissolution performance of the three formulations was sustained by their wetting behavior results (Table 6). When exposed to water, the L1 milk compressed samples showed a high contact angle, thus a low wetting capacity, as compared to the L3 milk samples, where the water spread immediately on their surface with a low contact angle and an improved surface free energy.

#### 3.4.2. Solid-State Analysis

**Figure 8:**
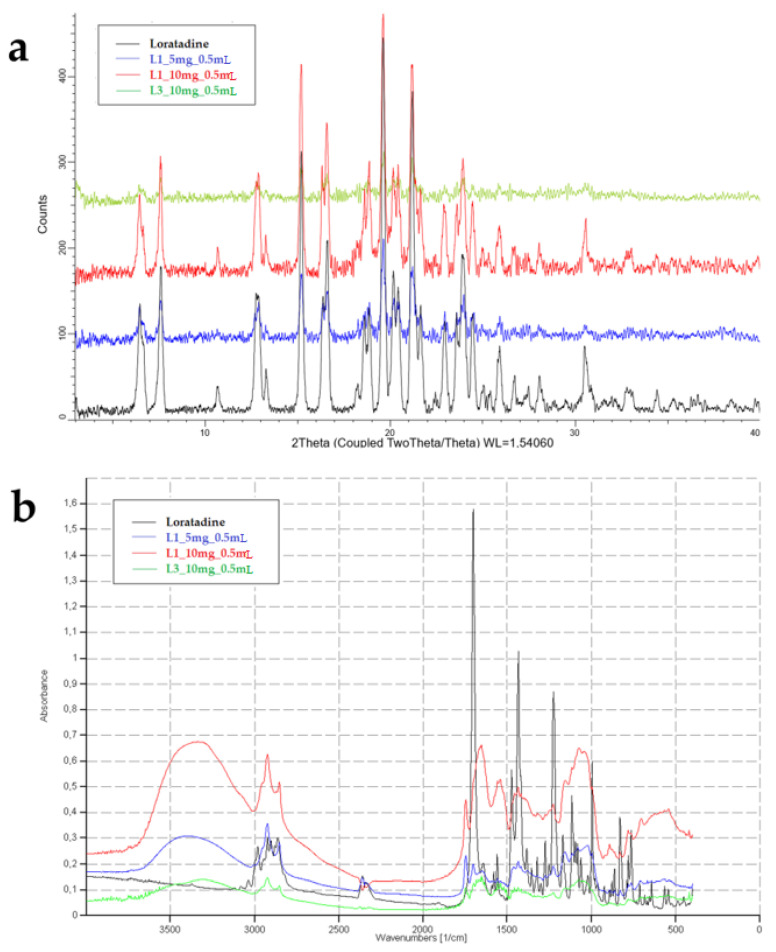
XRPD diffractograms (**a**) and FT-IR spectra (**b**) of raw loratadine and OL formulations.

As previous studies showed that loratadine dissolution behavior varied upon crystallinity [41], several determinations were made to assess the potential structural changes that may have occurred during OL preparation. Figure 8 displays the XRPD diffractograms and the FT-IR spectra of raw loratadine and selected formulations.

The XRPD diffractogram of raw loratadine showed its crystalline nature through numerous sharp peaks between 3 and 33 degrees of the 2-θ scale, at 12.7, 15.1, 16.5, 19.7, 21.3, 23.9, 30.5, and 32.5 degrees [42]. The overlapped patterns highlight the characteristic peaks of loratadine in the OL formulations. A similar pattern was obtained for L1_10 mg_0.5 mL, and the peaks were visible but less intense for L1_5 mg_0.5 mL, where the loratadine content was lower in favor of the milk fraction. However, the characteristic peaks of loratadine significantly faded in the diffractogram of L13_10 mg_0.5 mL, which revealed a decrease in crystallinity in the OL prepared using infant formula. These results that indicate the partial amorphization of loratadine explain the good wettability and dissolution results displayed by the L13_10 mg_0.5 mL formulation.

FT-IR analysis aimed to highlight the compatibility and interactions between loratadine and milk as an excipient by evaluating the changes that appeared in its structure after OL preparation compared to the pure substance. The characteristic FT-IR bands of loratadine were found at 997 cm^−1^ for aryl C-Cl stretching and at 1227 cm^−1^ for -C-N stretching. C=O bonds of the amide or ester groups were represented by bands at 1550 and 1703 cm^−1^ and showed the regions where intermolecular interaction could occur. Bands from 3000 to 2850 cm^−1^ revealed the C–H bond stretching [39]. The OL samples containing freeze-dried milk showed a characteristic band from 3468 to 3300 cm^−1^, which corresponded to O-H and N-H stretching in the protein structure, a single band at 1745 cm^−1^ for the C=O group stretching in the fatty esters, and the saccharide bands from 1200 to 900 cm^−1^ [21]. The increase in loratadine content that resulted in lower fractions of milk in the samples led to less intense signals of the milk constituents. All the characteristic peaks of loratadine were identified when included in the freeze-dried milk matrix, including the carbonyl band, but shifted to a lower wavenumber due to newly formed intermolecular hydrogen bonds [42]. As the FTIR results indicate the formation of new hydrogen bonding and no covalent bonds, the API shows good compatibility with the milk matrix [43].

**Figure 9:**
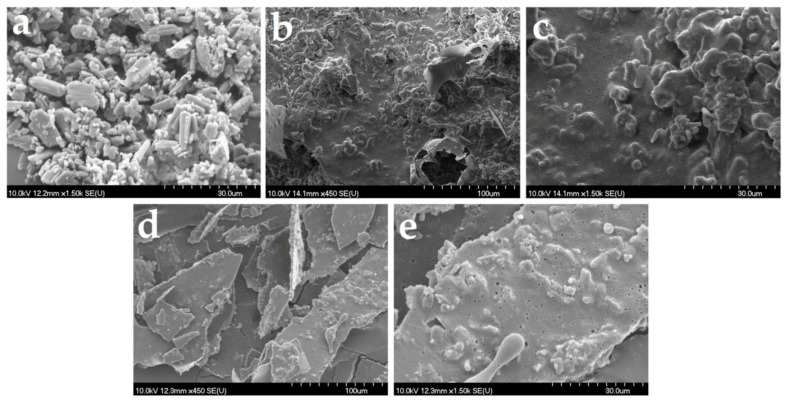
SEM images of raw loratadine (**a**) and OL formulations L1_10 mg_0.5 mL with 100 µm (**b**), 30 µm (**c**) magnitude, and L13_10 mg_0.5 mL with 100 µm (**d**), 30 µm (**e**) magnitude.

Raw loratadine crystals, presented as polydispersed rod-shaped or oval-shaped crystals, are shown in Figure 9a. The images in Figure 9b–e correspond to the formulations L1_10 mg_0.5 mL and L3_10 mg_0.5 mL, and images captured on the outer surfaces of the freeze-dried products focusing on the loratadine particles and on their appearance were included in the structures. In all images, API particles seem completely included in the pore walls and surrounded by the solid milk matrix. An agglomeration tendency was observed for the formulations with L1 milk, probably caused by its lower viscosity when compared to the one with L3 milk. Moreover, a slight reduction in particle size was visible, partially hindered by the agglomerations. However, this was not sustained by the particle size determinations, which indicates that despite the appearance of solid structures, they will easily and completely redisperse in water. L3 milk formulation displayed thicker pore walls when compared to L1 formulation; visible particles embedded into the pore walls but with lower sizes and better distribution throughout the solid matrix, with a lower agglomeration tendency due to the higher viscosity of the infant formula.

### 3.5. Practical Implications of the Study

A growing body of scientific literature is investigating the potential role of milk as a dispersing agent and drug carrier for poorly soluble drugs. Among liquid drug dispersions and compressed tablets, this study opens the way for the use of milk as an excipient for another dosage form: oral lyophilizate. However, even if the scientific evidence supports the performance and benefits of milk in drug development, certain aspects need to be considered. First, the regulatory hurdle, since milk is not registered as a pharmaceutical excipient. Its variability in the content of lipids and proteins is unlikely to lead to its approval as an excipient, as one of the main requirements is the consistency in composition and physical-chemical characteristics, sustained by the quality specification [24]. These drawbacks may be partially solved by using infant powdered formulae with a standardized composition [6], which, in the present study, gave the best results in terms of mechanical characteristics and API dissolution. Secondly, the potential allergic reactions should not be neglected, as bovine milk allergy represents the most common food allergy in infants and children [6,44], and furthermore, the potential unwanted interactions between milk compounds and drugs, such as the reaction of calcium ions with tetracycline [24]. In this respect, further studies are yet to be performed to obtain lactose-free formulations for lactose-intolerant patients [45] and to understand the features of the APIs that can be safely included into milk structures [24].

Of all the orodispersible forms studied using milk as an excipient, the products prepared in this study displayed a much faster disintegration in the context of adequate resistance. Therefore, lyophilization may be a viable option for the preparation of orodispersible milk-based products for substances that require ultra-fast release. Another key point of this study is the understanding of the influence of OL size and insoluble active substance loading on the characteristics of lyophilized structures. The obtained results could even be used to guide the development of oral lyophilizates with traditional excipients. Further studies could attempt to increase the strength of infant formula lyophilized matrices by adding small amounts of biocompatible polymers, investigate other infant formulae to understand how the properties of OLs are influenced by their composition, or aim at the loading of APIs with different physical-chemical properties in freeze-dried milk structures.

## 4. Conclusions

This study aimed at the preparation of freeze-dried orodispersible dosage forms out of milk as a matrix-forming agent. Bovine milk and infant formulae were first tested for their mechanical properties in their freeze-dried state. As they proved sufficient mechanical strength to withstand manipulation, they were loaded with an insoluble model API, loratadine, at increasing doses, to obtain OLs of different sizes, according to a DoE. The pharmaceutical properties of the prepared products fulfilled the official quality requirements for oral lyophilizates. They displayed acceptable disintegration using bovine milk and ultra-rapid disintegration using infant formulae. The mechanical parameters confirm the obtention of lyophilizates with sufficient resistance to handling for the formulae with bovine milk but also for infant formula L3, even when loading different doses of suspended active pharmaceutical ingredient.

## Figures and Tables

**Table 1:** Milk composition as declared by the producers.

	L1	L2	L3	L4
Proteins (g/100 mL)	3.1	3	1.27	1.2
Fat (g/100 mL)	1.5	3.5	3.4	3.6
- Saturated fatty acids (g/100 mL)	1	2.4	0.96	0.8
- Monounsaturated fatty acids (g/100 mL)	n.s.	n.s.	-	1.8
- Polyunsaturated fatty acids (g/100 mL)	n.s.	n.s.	-	0.6
Carbohydrates (g/100 mL)	4.5	4.5	5.13	7.4

n.s., not specified.

**Table 2:** Design of experiments matrix.

Experiment Name	Run Order	Loratadine Dose (X1)	Alveolae Volume (X2)	Milk Type (X3)
N1	4	0	0.2	L1
N2	16	10	0.2	L1
N3	19	0	1	L1
N4	15	10	1	L1
N5	14	0	0.2	L2
N6	11	10	0.2	L2
N7	8	0	1	L2
N8	18	10	1	L2
N9	10	0	0.2	L3
N10	13	10	0.2	L3
N11	9	0	1	L3
N12	6	10	1	L3
N13	7	0	0.2	L4
N14	5	10	0.2	L4
N15	3	0	1	L4
N16	2	10	1	L4
N17	12	5	0.5	L4
N18	1	5	0.5	L4
N19	17	5	0.5	L4

**Table 3:** Appearance, weight, and sizes of OLs obtained in different alveolae.

Alveolae Volume	0.2 mL	0.5 mL	1 mL
Aspect	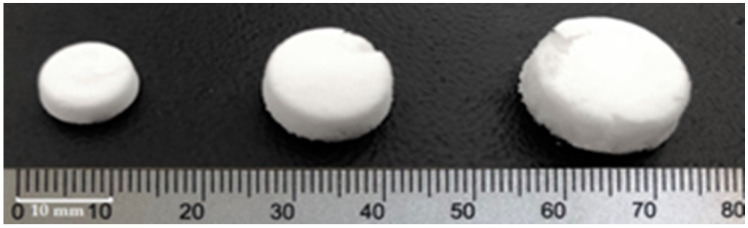		
Diameter (mm)	8.40 ± 0.35	11.82 ± 0.15	14.14 ± 0.85
Thickness (mm)	2.73 ± 0.19	4.02 ± 0.28	5.03 ± 0.24
Weight (g)	0.023 ± 0.002	0.056 ± 0.005	0.115 ± 0.010

**Table 4:** Model performance parameters for the DoE work set.

Response	R2	Q2	Validity	Reproducibility	ANOVA Regression	ANOVA Lack of Fit
Y1	0.993	0.982	0.756	0.992	0.000	0.378
Y2	0.927	0.899	0.501	0.983	0.000	0.136
Y3	0.871	0.779	0.640	0.944	0.000	0.237
Y4	0.798	0.555	0.720	0.862	0.001	0.326
Y5	0.671	0.431	0.300	0.966	0.007	0.061
Y6	0.780	0.618	0.435	0.960	0.001	0.105
Y7	0.822	0.574	0.642	0.922	0.000	0.240
Y8	0.888	0.621	0.972	0.568	0.001	0.895

Y1, disintegration time; Y2, hardness; Y3, rigidity; Y4, fracturability; Y5, % of dissolved loratadine after 5 min; Y6, % of dissolved loratadine after 10 min; Y7, particle size; Y8, PDI.

**Table 5:** Results of the DoE validation experiments.

Code		L1_5 mg_0.5 mL			L1_10 mg_0.5 mL			L13_10 mg_0.5 mL		
Formulation factors	X1	5			10			10		
	X2	0.5			0.5			0.5		
	X3	L1			L1			L3		
		Predicted	Actual	Residual	Predicted	Actual	Residual	Predicted	Actual	Residual
Responses	Y1	144.35	103.13 ± 22.57	−44.22	123.76	83.61 ± 16.28	−40.15	4.20	3.91 ± 0.45	−0.29
	Y2	1339.45	523.3 ± 160.20	−816.15	592.91	537.5 ± 100.03	−55.41	520.91	518.3 ± 20.70	−2.61
	Y3	700.70	122.2 ± 37.4	−578.5	223.70	118.3 ± 25.12	−105.4	196.99	127.00 ± 3.49	−69.99
	Y4	675.98	100 ± 38.4	−575.98	323.25	158.4 ± 50.17	−164.85	138.21	89.8 ± 5.84	−48.41
	Y5	28.05	31.40 ± 1.07	3.35	55.46	53.26 ± 7.08	−2.2	64.79	60.58 ± 4.11	−4.21
	Y6	34.68	47.79 ± 1.78	13.11	70.64	75.76 ± 11.36	5.12	77.12	89.09 ± 1.06	11.97
	Y7	359.14	355.07 ± 11.40	−4.07	386.79	390.90 ± 14.79	4.11	626.75	548.90 ± 3.48	−77.85
	Y8	0.375	0.370 ± 0.036	−0.005	0.463	0.453 ± 0.045	−0.01	0.385	0.327 ± 0.014	−0.058

Legend: L1_5 mg_0.5 ml—L1 type of milk, 0.5 mL sample volume, 5 mg loratadine; L1_10 mg_0.5 mL—L1 type of milk, 0.5 mL sample volume, 10 mg loratadine; L13_10 mg_0.5 mL—L3 type of milk, 0.5 mL sample volume, 10 mg loratadine.

**Table 6:** Wettability results.

	ΘWater [°]	ΘDiiodomethane [˚]	γ [mN m^−1^]	Polarity [%]
**Loratadine**	72.75	13.2	55.54	18.47
**L1_5 mg_0.5 mL**	71.46	1	57.14	18.62
**L1_10 mg_0.5 mL**	71.76	15	55.65	19.35
**L13_10 mg_0.5 mL**	1	10.2	82.22	45.16

Legend: L1_5 mg_0.5 mL—L1 type of milk, 0.5 mL sample volume, 5 mg loratadine; L1_10 mg_0.5 mL—L1 type of milk, 0.5 mL sample volume, 10 mg loratadine; L13_10 mg_0.5 mL—L3 type of milk, 0.5 mL sample volume, 10 mg loratadine.

## Data Availability

Not applicable.

## Supplementary Materials

The following supporting information can be downloaded at: www.mdpi.com/article/10.3390/pharmaceutics14071342/s1, Table S1. Response matrix.
