# Supplementary material for: Milk Oral Lyophilizates with Loratadine: Screening for New Excipients for Pediatric Use
Source: Pharmaceutics. 2022 Jun 24;14(7):1342. doi: 10.3390/pharmaceutics14071342 (PMC9318030; doi:10.3390/pharmaceutics14071342)
Supplement: Supplementary file 1 [file pharmaceutics-14-01342-s001.zip › pharmaceutics-1761794-supplementary.pdf]

Table S1. Response matrix

	Y1	Y2	Y3	Y4	Y5	Y6	Y7	Y8
N1	149.03 ± 15.96	2198 ± 313.2	1774 ± 13.4	1524 ± 21.58	0	0	318.83 ± 3.81	0.292 ± 0.043
N2	109.15 ± 12.81	657.7 ± 113.3	208.7 ± 27.72	548.5 ± 129.9	67.16 ± 3.97	73.22 ± 2.33	444.2 ± 18.54	0.524 ± 0.055
N3	235.72 ± 8.78	4160 ± 153.3	3108 ± 197.3	1458 ± 37.96	0	0	292.97 ± 6.67	0.265 ± 0.007
N4	133.56 ± 10.36	654.5 ± 79.6	277 ± 38.25	240.7 ± 67.3	39.65 ± 0.82	63.06 ± 3.02	359.6 ± 4.31	0.386 ± 0.042
N5	163.7 ± 11.07	2424 ± 468.5	1712 ± 346.3	989.7 ± 51.17	0	0	458.23 ± 13.25	0.459 ± 0.067
N6	139.48 ± 12.15	695 ± 67.15	292.3 ± 31.2	169.7 ± 19.5	36.49 ± 1.75	52.06 ± 1.80	567.27 ± 34.13	0.531 ± 0.053
N7	265.39 ± 7.72	4486 ± 44.9	3735 ± 253.5	3320 ± 64.09	0	0	401.07 ± 5.35	0.352 ± 0.041
N8	162.41 ± 8.16	762.8 ± 82.5	313.2 ± 55.5	423.7 ± 38.25	25.51 ± 0.41	54.45 ± 2.82	459.5 ± 14.46	0.463 ± 0.018
N9	4.61 ± 1.22	2786 ± 529.3	1882 ± 408.8	257.3 ± 7.44	0	0	665.83 ± 34.9	0.333 ± 0.044
N10	4.06 ± 0.53	412.3 ± 26.5	164.3 ± 19	236.3 ± 17.62	88.07 ± 1.65	99.77 ± 0.33	535.47 ± 15.33	0.376 ± 0.073
N11	8.39 ± 0.85	4205 ± 352.4	3438 ± 598.8	1427 ± 82.74	0	0	539.17 ± 15.17	0.294 ± 0.044
N12	4.31 ± 0.87	485.5 ± 46.1	180.3 ± 30.53	113 ± 9.15	56.06 ± 4.10	62.44 ± 3.38	634.97 ± 21.20	0.377 ± 0.071
N13	1.37 ± 0.11	1317 ± 209.4	1008 ± 156.8	210 ± 17.2	0	0	516.63 ± 21.7	0.35 ± 0.022
N14	1.48 ± 0.35	289.8 ± 11.24	82.2 ± 2.73	42.2 ± 1.63	57.07 ± 5.78	70.27 ± 3.96	538.93 ± 9.87	0.384 ± 0.029
N15	8.09 ± 0.72	2065 ± 45.05	1330 ± 55.22	863 ± 24.11	0	0	456.1 ± 13.65	0.26 ± 0.043
N16	4.06 ± 0.82	520 ± 13.3	276.5 ± 36	131.2 ± 14.48	68.46 ± 4.45	99.18 ± 0.29	551.37 ± 10.84	0.316 ± 0.047
N17	3.79 ± 0.16	410.7 ± 28.3	102.8 ± 11.65	37.5 ± 1.69	103.76 ± 7.14	102 ± 1.41	510.5 ± 9.76	0.267 ± 0.047
N18	4.5 ± 0.57	460 ± 32.16	70 ± 8.89	20 ± 2.21	97.62 ± 5.72	100 ± 6.49	456.7 ± 7.71	0.374 ± 0.028
N19	3.2 ± 0.39	360 ± 38.56	132 ± 14.22	57.5 ± 6.41	89.52 ± 1.69	90.81 ± 5.96	487.6 ± 7.28	0.314 ± 0.034

Y1, disintegration time; Y2, hardness; Y3, rigidity; Y4, fracturability; Y5, % of dissolved loratadine after 5 minutes; Y6, % of dissolved loratadine after 10 minutes; Y7, particle size; Y8, PDI
